# Sex-Related Motor Deficits in the Tau-P301L Mouse Model

**DOI:** 10.3390/biomedicines9091160

**Published:** 2021-09-04

**Authors:** Luana Cristina Camargo, Dominik Honold, Robert Bauer, N. Jon Shah, Karl-Josef Langen, Dieter Willbold, Janine Kutzsche, Antje Willuweit, Sarah Schemmert

**Affiliations:** 1 Institute of Biological Information Processing, Structural Biochemistry (IBI-7), Forschungszentrum Jülich, 52425 Jülich, Germany; l.camargo@fz-juelich.de (L.C.C.); d.honold@fz-juelich.de (D.H.); Robert.Bauer@hhu.de (R.B.); d.willbold@fz-juelich.de (D.W.); j.kutzsche@fz-juelich.de (J.K.); 2 Institut für Physikalische Biologie, Heinrich-Heine-Universität Düsseldorf, 40225 Düsseldorf, Germany; 3 Institute of Neuroscience and Medicine, Medical Imaging Physics (INM-4), Forschungszentrum Jülich, 52425 Jülich, Germany; n.j.shah@fz-juelich.de (N.J.S.); k.j.langen@fz-juelich.de (K.-J.L.); a.willuweit@fz-juelich.de (A.W.); 4 JARA-Brain-Translational Medicine, JARA Institute Molecular Neuroscience and Neuroimaging, 52062 Aachen, Germany; 5 Department of Neurology, RWTH Aachen University, 52062 Aachen, Germany; 6 Department of Nuclear Medicine, RWTH Aachen University, 52062 Aachen, Germany

**Keywords:** tauopathy, Tau-P301L mouse models, behavior, phosphorylated Tau, motor deficits, cognitive deficits, sex-related deficits

## Abstract

The contribution of mouse models for basic and translational research at different levels is important to understand neurodegenerative diseases, including tauopathies, by studying the alterations in the corresponding mouse models in detail. Moreover, several studies demonstrated that pathological as well as behavioral changes are influenced by the sex. For this purpose, we performed an in-depth characterization of the behavioral alterations in the transgenic Tau-P301L mouse model. Sex-matched wild type and homozygous Tau-P301L mice were tested in a battery of behavioral tests at different ages. Tau-P301L male mice showed olfactory and motor deficits as well as increased Tau pathology, which was not observed in Tau-P301L female mice. Both Tau-P301L male and female mice had phenotypic alterations in the SHIRPA test battery and cognitive deficits in the novel object recognition test. This study demonstrated that Tau-P301L mice have phenotypic alterations, which are in line with the histological changes and with a sex-dependent performance in those tests. Summarized, the Tau-P301L mouse model shows phenotypic alterations due to the presence of neurofibrillary tangles in the brain.

## 1. Introduction

Tau protein is a microtubule associated protein, located in the axons, which plays a major role in the stabilization of microtubules [1] and trafficking [2,3,4]. It is expressed by the microtubule-associated protein Tau (*MAPT*) gene located on the chromosome 17. In total, six isoforms can be produced by the presence/absence of exon 2, 3 (N-terminal) and 10 (microtubule-binding domain). Therefore, the isoform expression varies from 0N3R, which is the shortest form, to 2N4R, which is the longest form. In humans, the 3R is more frequent during the development, while both 3R and 4R, are present in similar amount in the adult brain [5,6]. Phosphorylation of the Tau protein can occur at different sites by different kinases, a process that assists in Tau physiological function. Under pathological conditions, the Tau binding site to the microtubules is hyperphosphorylated and results in loss of its function. Hyperphosphorylated Tau then assembles into paired helical filament (PHF) forming the neurofibrillary tangles (NFTs) in the dendrites [6,7]. Pathological Tau is present in different neurodegenerative diseases called tauopathies.

Tauopathies, in turn, are a heterogeneous class of diseases that can be classified as primary and secondary tauopathies. In secondary tauopathies, the presence of NFTs occurs as a second event probably due to the toxicity downstream of another event, e.g., aggregation of amyloid-β (Aβ) into neuritic plaques in Alzheimer’s disease (AD). In primary tauopathies, the presence of NFTs occurs first and is mainly responsible for the arising neurodegeneration, e.g., in frontotemporal dementia (FTD) [8]. In those dementias, the formation of NFTs in a specific region is correlated with progression of the disease and brain atrophy [9,10]. Considering that brain atrophy and cognitive deficits are a consequence of neurodegeneration and synaptic dystrophy, it is postulated that the presence of NFTs induces synaptic deficits and neurodegeneration [11,12]. Besides in dementias, pathological Tau can also be found in patients with epilepsy, chronic traumatic encephalopathy and other neurological disorders [13]. Similar to AD, most of the FTDs and other tauopathies are sporadic and, unlike AD, different mutations can cause the familial FTDs. The mutations in the *MAPT* gene are genetic causes of FTDs with parkinsonism linked to chromosome 17 (FTDP-17) [14,15]. Those mutations prevent Tau from binding to microtubules due to hyperphosphorylation [16]. 

Many transgenic mouse models have been developed with different Tau mutations. Those models provide a more detailed understanding of how hyperphosphorylated Tau and NFTs affect the pathophysiology, depending on the type of mutation and the isoform. The most common transgenic models of tauopathy are constructed with the human Tau-P301L mutation [17,18]. The Tau-P301L mouse models only include the 4R Tau isoform, since this mutation is located in the exon 10. Terwel and collaborators [19] developed a transgenic mouse model expressing human Tau-P301L (homozygous) under the regulation of a *thy1* gene promoter at moderate levels. This mouse model did not develop severe motor deficits, but a strong paralysis in the limbs, starting at nine months of age. The mice died before the age of 12 months due to respiratory problems [19,20]. Moreover, Tau-P301L mice showed NFTs at nine months of age in the brainstem and cortex [19]. The presence of NFTs in different areas of the brainstem was postulated to be the cause of respiratory deficits and strong moribund conditions [20]. At earlier ages, this mouse model also showed increased long-term potentiation (LTP) in the dentate gyrus (DG) [21].

Nowadays, mouse models are considered a method to represent human diseases and to test newly developed substances as treatment. Mouse models, especially for neurodegenerative diseases, have recently been under some criticism, in part because many clinical trials failed even though the compounds did previously show promising results in animal models. Very often in these cases, however, treatment studies in mice often had an insufficient study design, which does not mimic the human situation very well. It is essential to know your animal model as well as possible, especially concerning the selection of behavioral tests and to characterize them in longitudinal studies, instead of just analyzing deficits at one specific age, as well as doing this in a sex-specific manner. The objective of this study was to carry out a longitudinal and sex-related characterization of the Tau-P301L model to clarify the onset of the disease with a broader behavioral test battery and to have an in-depth understanding about the deficits of the model. As described before, the Tau-P301L model was evaluated in few behavioral experiments (beam walk, rotarod and novel object recognition) and some studies were cross-sectional. A longitudinal study is advantageous since the onsets of each behavioral deficit occur at different time points; therefore, the cross-sectional studies have limited information regarding the course of the disease. Thus, the present study focused on the characterization of general, motor and cognitive alterations induced by pathological Tau in the Tau-P301L mouse model at different ages and sexes.

## 2. Materials and Methods

### 2.1. Animals

Tau-P301L mice were first described by Terwel et al. [19] and were backcrossed from a FVB to a C57BL/6J background. Mice were maintained in a homozygous colony. In this study, we compared homozygous Tau-P301L mice with age- and sex-matched wild type (WT) mice from a parallel breeding.

Mice were bred in-house with a 12/12 h light/dark cycle. In each cage, three to five mice were housed and food and water were available *ad libitum*. All behavioral experiments were approved by the responsible authorities (*Landesamt für Natur, Umwelt und Verbraucherschutz (LANUV)*, North Rhine-Westphalia, Germany, number 84-02.04.2014.A362, 81-02.04.2018.A400 and 81-02.04.2019.A304; approval was received on 05/02/2019, 21/02/2019 and 21/01/2019, respectively) and were performed longitudinally at different ages (2, 4, 6 and 8 months). For all behavioral tests, 7 female and 12 male mice of both genotypes were included. 

### 2.2. Behavioral Tests 

#### 2.2.1. Habituation/Dishabituation Olfactory Test

Olfactory deficits from Tau-P301L mice were evaluated by performing the habituation/dishabituation olfactory test [22]. Three different aromas (bacon, cheesecake and hazelnut) (Perfumer’s Apprentice, Scotts Valley, CA, USA) were sprayed on a cotton pad which was placed into an embedding cassette. The bacon aroma was placed in the cage for 24 h before the test for habituation. Later, the bacon aroma was presented again to the mice for six times for 30 s each. Next, the bacon aroma was replaced by cheesecake and hazelnut aroma once (30 s each). The time the mice sniffed each embedding cassette was recorded for analysis.

#### 2.2.2. Nesting Behavior Test

Nesting behavior was performed as previously described [23]. One hour before the dark cycle of the animal facility, the mice were single caged with new nesting material. The next morning, the built nest was scored from 1 to 5, whereby 1 was no nest and 5 was a fully built nest. 

#### 2.2.3. Marble Burying Test

In the marble burying test [24], mice were placed in a cage with 5 cm of bedding material with 12 equally distant marbles for 30 min, which were placed on the top of the bedding material. Later, the mice were placed back in the habituation cage and the number of marbles each mouse had buried was counted for analysis. 

#### 2.2.4. SHIRPA Test Battery

To evaluate the phenotypic alterations of Tau-P301L mice in comparison to the WT mice, the SmithKline Beecham Pharmaceuticals; Harwell, MRC Mouse Genome Centre and Mammalian Genetics Unit; Imperial College School of Medicine at St Mary’s; Royal London Hospital, St Bartholomew’s and the Royal London School of Medicine; Phenotype Assessment (SHIRPA)-test battery was performed (protocol adapted from [25]). In this test, the different parameters described in Table 1 were evaluated in a scoring system from 0 to 3 (0 = no alteration; 1 = slightly altered; 2 = altered; 3 = strongly altered). 

#### 2.2.5. Open Field Test

In the open field test, mice were placed in a cubicle arena (40 cm) for 30 min. During this time, mice were allowed to freely explore the arena, imaginarily divided into different zones (border, center, corner). For evaluation, tracking software was used (EthoVision XT15, Noldus Information Technology, Wageningen, The Netherlands). The following parameters were analyzed: velocity, locomotion, exploration time, time spent in center, border and corner zone.

#### 2.2.6. Accelerating Rotarod

The accelerating Rotarod (Ugo Basile, Gemonio, Italy) test consisted of four trials. In the first trial, the mice were placed onto the rod and should stay there for at least 60 s at 10 rpm (habituation to the apparatus). If they fell, the trial was repeated. In the last three trials, the mice should stay on the rod for 300 s at 4 to 40 rpm. For evaluation, the latency time to fall was noted and the mice were placed back into their home cages. Three sessions in each trial with an interval of 15 min were performed [26].

#### 2.2.7. Modified Pole Test

In order to gain a deeper understanding of the developed motor deficits, a modified version of the so-called pole test was performed [27]. For this, mice were placed facing down on the top of a pole and the way they walked down was scored three times. The scoring system was: 0 = running, 1 = partly running, 2 = slipped and 3 = fallen. This procedure was repeated three times with an interval of 15 min between each trial. For the final evaluation, the sum of the three scores was calculated.

#### 2.2.8. Novel Object Recognition Test

For the novel object recognition test (NOR), two identical objects (familiar object) were presented to the mice during 10 min in the same arena used for the open field test. In the inter-trial interval of 20 min, the mice were placed back in their home cages. Afterwards, the mice were placed back into the arena where one familiar object was replaced by a new object (novel object). The time of exploration was evaluated as the time the mouse spent with the nose at least 2 cm from the object. This was analyzed by EthoVision XT15 (Noldus Information Technology, Wageningen, The Netherlands). 

For evaluation, the discrimination index was calculated by the following formula:

(1) $$\frac{Tnovel-Tfamiliar}{Tnovel+Tfamiliar}$$

where *Tnovel* was the time the mice explored the novel object and *Tfamiliar* was the time the mice explored the familiar object. 

#### 2.2.9. T-Maze Spontaneous Alternation

In the T- maze spontaneous alternation [28], the mice were placed in the starting arm in an arena with three arms (start, left and right arm) (31 cm × 10 cm) in a “T” format. In the first trial, only the left or right arm was free to be explored and the opposite one was closed by a gate. Once the mice came back to the start arm, both arms were free to be explored and the second trial started. The same procedure was performed for 14 trials or a maximum of 15 min. If a mouse did not reach seven trials, it was excluded from the experiment.

The spontaneous alternation was calculated by the following formula:

(2) $$\frac{number\;of\;correct\;choices}{total\;of\;trials}$$

Correct choices are considered as interactions with the arm opposite to the one that the mouse previously entered in the maze. 

#### 2.2.10. Fear Conditioning Test

In order to evaluate the associative memory deficits, the cued and contextual fear conditioning was performed [29] starting with 4 months of age. On the habituation day, mice were placed in the apparatus (Ugo Basile, Gemonio, Italy) for 120 s of habituation. Afterwards, a sound (50%; 2000 Hz) was presented for 30 s, and during the last 2 s, a mild shock (0.35 mA) was also given. The mice stayed in the cage for additional 60 s before returning to their home cages. 

The next day, the contextual fear conditioning was evaluated. The mice were placed in the same cage for 5 min and neither the shock nor the sound were presented. After 25 min, the cued fear conditioning was evaluated. The walls and floor of the cage were changed and only the sound was presented three times to the mice. The freezing (%) was analyzed with tracking software (EthoVision XT15, Noldus Information Technology, Wageningen, the Netherlands).

#### 2.2.11. Morris Water Maze

The performance of the Morris water maze (MWM) [30] was divided into 3 stages: training, probe and reversal test. For the MWM training, the mice were placed in a pool (diameter of 120 cm × 60 cm height) filled with water divided into 4 quadrants (NE, NW, SE, SW) with a hidden platform (diameter of 10 cm × 31.5 cm height). An opaque, non-toxic liquid was added into the water to prevent the mice from seeing the platform. For a maximum of 60 s, the mice had to find the hidden platform. In case the mice did not find it, they were placed onto the platform for 10 s for acquisition (to orientate themselves). This trial was then repeated four times per mouse. Additionally, at each trial, the mice were placed in a different starting position. These trials were performed for four consecutive days. On the fifth day, the platform was removed and the probe trial was performed. Moreover, the reversal test was also performed, similar to the training, for three consecutive days and the platform was placed in a different position (opposite position). Similar to the previous cognitive tests, the evaluation and tracking was analyzed by tracking software (EthoVision XT15, Noldus Information Technology, Wageningen, The Netherlands). In the training and reversal test, the time the mice needed to find the hidden platform (escape latency) was analyzed. In the probe trial, the time spent in the platform zone was analyzed. The MWM was performed only at 8 months of age. One female mouse developed a forelimb paralysis and was, therefore, excluded from the MWM experiment.

### 2.3. Histology

After the performance of the last behavioral tests (MWM), mice were deeply anesthetized for tissue collection. The brains were snap frozen and one hemisphere was cut into 20 µm sagittal sections using a Cryotome (Leica Biosystems Nussloch GmbH, Wetzlar, Germany). Before the staining procedure, the brain slices were placed in 4% formalin and washed three times with TBS for 5 min. Antigen retrieval was performed in citrate buffer, pH 6 at 85 °C for 30 min and slides were washed thre times with TBS for 5 min. In order to remove the endogenous peroxidases, the sections were incubated in 0.6% H_2_O_2_ in methanol for 15 min and washed once with deionized water and two times with TBS for 5 min. Then, the sections were blocked in 10% horse serum for 1 h and incubated overnight with the primary antibody (AT8 (1:500; MN1020, Thermo Fisher scientific, Waltham, MA, USA) or AT100 (1:500; MN1060, Thermo Fisher scientific, Waltham, MA, USA) in 1% horse serum in TBS at 4 °C. On the subsequent day, the sections were washed and incubated with the secondary antibody (biotinylated goat anti-mouse, 1:1000; Extra2, Sigma-Aldrich, Darmstadt, Germany) for 2 h. Afterwards, slides were again washed and incubated with ExtrAvidin^®^ (1:1000; Extra2, Sigma-Aldrich, Darmstadt, Germany) for additional 2 h, followed by a washing step. Finally, the sections were colored with DAB and saturated nickel ammonium sulphate solution, washed, dehydrated in an ascending alcohol series and mounted with DPX (Sigma-Aldrich, Darmstadt, Germany). 

To evaluate neurodegeneration and neuroinflammation, the following staining procedure was done. The brain slides were placed in 4% formalin and washed three times with TBS-T (1% triton) for 5 min. Antigen retrieval was performed in 70% formic acid and slides were washed. In order to remove the endogenous peroxidases, the sections were incubated in 3% H_2_O_2_ in methanol solution for 15 min and washed. Then, the sections were incubated overnight with the primary antibody (NeuN (1:1000; Merck, Darmstadt, Germany) and GFAP (1:1000; MN1060, Thermo Fisher scientific, Waltham, MA, USA) in 3% BSA in TBS-T at 4 °C. The next day, the sections were washed and incubated with the secondary antibody (biotinylated goat anti-rabbit, 1:1000; Thermo Fisher scientific, Dreieich, Germany) for 2 h. Afterwards, the same procedure was performed as described above. For the detection of reactive microglia (CD11b, 1:2000, Abcam, Berlin, Germany), the staining procedure was the same as previously described although the primary antibody was incubated in 1% normal goat serum (NGS) and 1% bovine serum albumin (BSA) at room temperature for 1.5 h and the washing buffer was TBS. Subsequently, the same procedure was performed as described above. 

The images were taken with a LMD6000 microscope and a DFC310 FX camera (Leica Biosystems Nussloch GmbH, Wetzlar, Germany) or with a Zeiss SteREO Lumar V12 microscope and the according software (Zeiss AxioVision 6.4 RE). For pathological Tau, the positive signals in the brainstem (hind and midbrain), cerebellum and cortex were counted with ImageJ software (National Institute of Health, Bethesda, MD, USA). For neuronal death, the positive signals in the brainstem (hind and midbrain), cerebellum and cortex were counted with Cell profiler software (Broad Institute, Cambridge, MA, USA). For reactive microglia and reactive astrocyte analysis, the stained areas (percentage) in the brainstem (hind and midbrain), cerebellum and cortex were analyzed with CellProfiler software (Broad Institute, Cambridge, MA, USA). For each staining, eight males and seven females were analyzed and four to eight slides were taken per mouse for analysis.

### 2.4. Statistical Analysis

The statistical analyses were performed using GraphPad Prism 8.3 (GraphPad Software, San Diego, CA, USA). Two-way ANOVA and Sidak’s multiple comparison post hoc were used as statistical analysis to compare the sex-matched WT with Tau-P301L mice at each age in all behavioral tests, except the habituation/dishabituation olfactory test, the NOR and the MWM. In the habituation/dishabituation olfactory test, the two-way ANOVA and Sidak’s multiple comparison post hoc was performed to compared the WT with Tau-P301L mice for each odor presented. In the NOR, the discrimination index was compared against the theoretical mean of 0%, which means the exploration of novel and familiar object are similar; therefore, no discrimination is assumed, and the statistical analysis was calculated by the one-sample T-test for each group. In the MWM, the two-way ANOVA analysis was performed to compare the WT males, WT females, Tau-P310L male and female mice during the training days in both, the training test and the reversal trial. In the MWM probe trial, the two-way ANOVA and Tukey’s multiple comparison post hoc was performed to analyze the difference in the time spent at each quadrant. To evaluate the differences in the histology, the two-way ANOVA and Sidak’s multiple comparison post hoc was used to compare the males to females and to compare the Tau-P301L to the WT. 

## 3. Results

### 3.1. Tau-P301L Male Mice Show Phenotypic Alterations Beginning at 4 Months of Age

**Figure 1:**
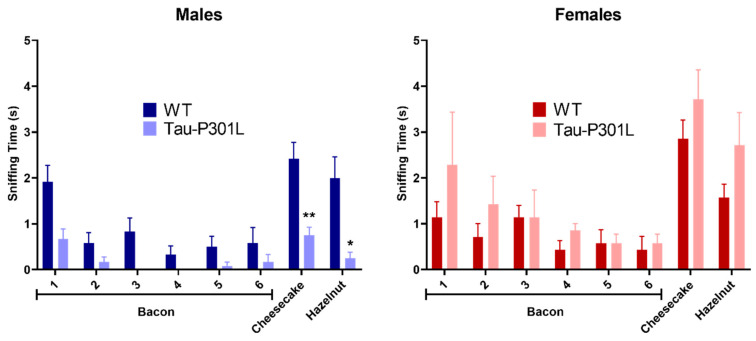
Tau-P301L mice develop olfactory deficits in the habituation/dishabituation olfactory test at 6 months of age. The bacon aroma was presented six times to the mice, the cheesecake and hazelnut aroma were presented afterwards. The sniffing (exploration) time was evaluated as the time the mouse placed the nose on the box with aroma-sprayed cotton. Tau-P301L male mice (*n* = 12) smelled the new aroma less (cheesecake and hazelnut) compared to the age-matched WT male mice (*n* = 12), but this was not observed in the females (*n* = 7). The two-way ANOVA was used as statistical analysis. *: *p* < 0.05 and **: *p* < 0.01 compared to the age-matched WT. Data are given as mean ±SEM.

In order to analyze the phenotypic alterations of male and female Tau-P301L mice, several behavioral tests were performed. Due to the fast-phenotypic progression, the Tau-P301L mice were regularly observed (at least once a week) and observations were reported in a score sheet. Regarding the general behavior, neither male nor female Tau-P301L mice showed any abnormalities in their home cages until 7 months of age. From 7 months of age, Tau-P301L mice progressed to a prominent paralysis of the limbs, and consequently, a loss of body weight and reduced movement in the home cages, as Terwel and collaborator [19] described. By comparison of the body weight, Tau-P301L mice had similar weight compared to WT mice, although Tau-P301L female mice had a slightly higher weight compared to WT female mice at 4 months of age (Figure S1). Tau-P301L mice did not display any deficits in the nesting behavior and marble burying compared to WT mice at all analyzed ages (Figure S2). Moreover, WT male mice buried a smaller number of marbles throughout aging. Furthermore, Tau-P301L mice had a non-significant trend of burying less marbles throughout aging (Figure S2). In the habituation/dishabituation olfactory test, Tau-P301L male mice explored the novel aroma less (cheesecake and hazelnut aroma) compared to the male WT mice at six months of age (Figure 1) (two-way ANOVA, *p* = 0.0036, *p* = 0.0139, respectively). Tau-P301L male mice were not able to discriminate the cheesecake and hazelnut from the bacon aroma. At 6 months of age, unlike Tau-P301L male mice, the Tau-P301L female mice did not show any olfactory deficits (Figure 1). 

**Figure 2:**
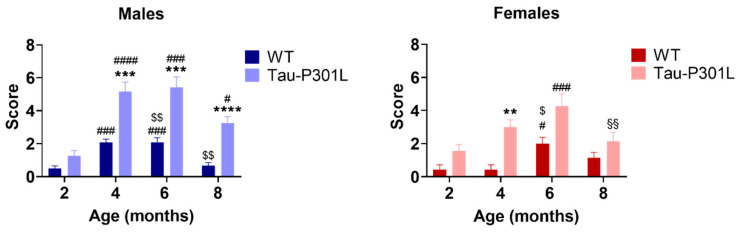
Tau-P301L male mice show phenotypic alteration in the SHIRPA test battery. Both Tau-P301L mice and WT were evaluated at 2, 4, 6 and 8 months of age. At 4, 6 and 8 months of age, Tau-P301L male mice (*n* = 12) had a higher score compared to the age-matched WT male mice (*n* = 12). Only at 4 months of age, Tau-P301L female mice (*n* = 7) had a higher score compared to the age-matched WT female mice (*n* = 7). Two-way ANOVA was performed. **: *p* < 0.01, ***: *p* < 0.001 and ****: *p* < 0.0001 compared to the age-matched WT. #: *p* < 0.05, ###: *p* < 0.001 and ####: *p* < 0.0001 compared to 2 months, genotype-matched. $: *p* < 0.05 and $$: *p* < 0.01 compared to 4 months, genotype-matched. §§: *p* < 0.01 compared to 6 months, genotype-matched. Data are given as mean ±SEM.

In the SHIRPA test battery, Tau-P301L male and female mice had phenotypic alterations compared to the sex-matched WT mice starting at 4 months of age (Table 2). Tau-P301L mice showed an abnormal gait, as demonstrated by a waddling walk. Moreover, they appeared to be less agile; they were slower than WT mice at 8 months of age. Furthermore, Tau-P301L mice showed an abnormal body carriage (hunched back) compared to WT male mice starting at 4 months of age. When lifted up by the tail, Tau-P301L mice presented clasping of all limbs, especially with increasing age, which can be described as slight paralysis starting at 6 months of age. This paralysis increased dramatically with age. Those findings are in correspondence with those published by Terwel et al. [19]. When placed hanging on a rod, Tau-P301L mice were not able to hold nor hang with both forelimbs starting with 4 months of age, but some WT mice showed similar impairments at 6 months of age. Finally, some Tau-P301L mice showed a mild loss of postural reflex when placed on their back starting with 4 months of age (Table 2; Table S1). Tau-P301L mice had higher SHIRPA scores compared to WT mice from 4 months onward (Figure 2) (two-way ANOVA; 4 months: *p* = 0.0008, 6 months: *p* = 0.0009, 8 months: *p* < 0.0001). Moreover, an age-dependent deterioration of the phenotype was observed starting at 2 months of age (two-way ANOVA; 2 vs. 4: *p* < 0.0001; 2 vs. 6: *p* = 0.0003; 2 vs. 8: *p* = 0.0204). Tau-P301L female mice had a higher score compared to WT female mice at 4 months of age (two-way ANOVA; *p* = 0.0022) as well as an increased score compared to 2 and 6 months (two-way ANOVA; 2 vs. 6: *p* = 0.0002; 6 vs. 8: *p* = 0.0030) (Figure 2).

### 3.2. Tau-P301L Male Mice Display Early Motor Deficits

**Figure 3:**
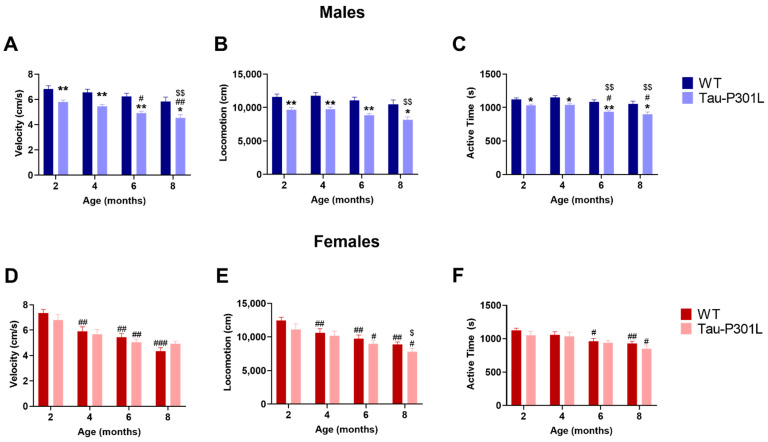
Tau-P301L male mice develop motor deficits in the open field test. Both Tau-P301L mice and WT were evaluated at 2, 4, 6 and 8 months of age. At all ages, Tau-P301L male mice (*n* = 12) were slower (**A**), travelled less (**B**) and were less active (**C**) compared to the age-matched WT males (*n* = 12). Tau-P301L female mice (*n* = 7) had similar velocity (**D**), locomotion (**E**) and active time (**F**) compared to the age-matched WT females (*n* = 7). Two-way ANOVA was performed. *: *p* < 0.05 and **: *p* < 0.01 compared to the age-matched WT. #: *p* < 0.05, ##: *p* < 0.01 and ###: *p* < 0.001 compared to 2 months genotype-matched. $: *p* < 0.05 and $$: *p* < 0.01 compared to 4 months genotype-matched. Data are given as mean ±SEM.

In the open field test, Tau-P301L male mice had motor deficits from to 2 months of age, since they were slower (two-way ANOVA; 2 months: *p* = 0.0068; 4 months: *p* = 0.0059; 6 months: *p* = 0.0015; 8 months: *p* = 0.0298) (Figure 3A) and travelled less (two-way ANOVA; 2 months: *p* = 0.0073; 4 months: *p* = 0.0060; 6 months: *p* = 0.0020; 8 months: *p* = 0.0296) (Figure 3B). This deficit persisted until 8 months of age and progressed throughout aging (two-way ANOVA; 2 vs. 6: *p* = 0.0003; 2 vs. 8: *p* = 0.0003; 4 vs. 8: *p* = 0.0031). Regarding the exploratory behavior, Tau-P301L male mice also spent less time exploring the arena compared to WT male mice beginning at 2 months of age (two-way ANOVA; 2 months: *p* = 0.0216; 4 months: *p* = 0.0198; 6 months: *p* = 0.0011; 8 months: *p* = 0.0454) (Figure 3C). Mice of both genotypes spent the same amount of time in the corner, border and center zone of the arena, demonstrating that Tau-P301L mice do not have increased anxiety levels compared to WT mice (Figure S4). Similar to the previous data, Tau-P301L female mice did not show any differences compared to the WT female mice, although both Tau-P301L and WT female mice showed a decrease of velocity, distance travelled and active time with aging (Figure 3D–F).

**Figure 4:**
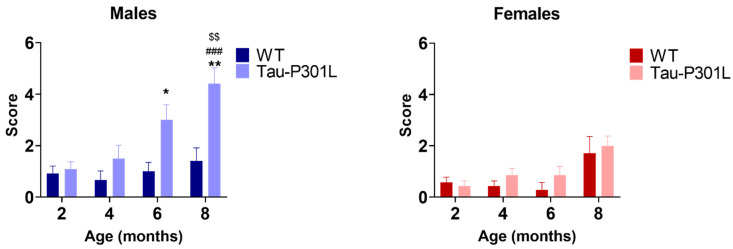
Tau-P301L male mice displayed motor deficits in the modified pole test. Both Tau-P301L mice and WT mice were evaluated at 2, 4, 6 and 8 months of age. The test was performed three times with a 15 min intertrial interval and the sum of the three trials was used for analysis. At 6 and 8 months of age, Tau-P301L male mice (*n* = 12) had a higher score compared to the age-matched WT male mice (*n* = 12), but this was not observed in the females (*n* = 7). Two-way ANOVA was performed. *: *p* < 0.05 and **: *p* < 0.01 compared to the age-matched WT. ###: *p* < 0.001 compared to 2 months genotype-matched. $$: *p* < 0.01 compared to 4 months genotype-matched. Data are given as mean ±SEM.

Analysis of the modified pole test revealed that Tau-P301L male mice had higher scores compared to the WT male mice starting at 6 months of age (Figure 4) (two-way ANOVA; 6 months: *p* = 0.0365 and 8 months: *p* = 0.0040). This indicates that Tau-P301L mice developed motor deficits in this test and the deficits progressed throughout aging (two-way ANOVA; 2 vs. 8: *p* = 0.0003; 4 vs. 8: *p* = 0.0031) (Figure 4). Tau-P301L female mice had similar performance as the WT female mice, indicating no motor deficits in this test.

Analysis of the Rotarod performance of both Tau-P301L male and female mice did not show any motor alteration in the accelerating Rotarod (Figure S3). Similar to the previously described paralysis, these results are in correspondence with those published by Terwel et al. [19].

### 3.3. Tau-P301L Show Mild Cognitive Deficits in the NOR

**Figure 5:**
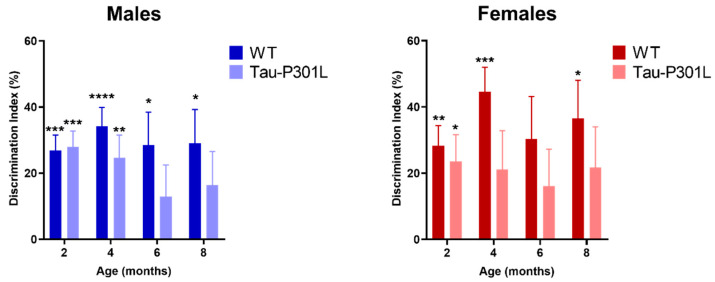
Deficits in recognition memory in Tau-P301L mice in the novel object recognition test (NOR). Both Tau-P301L mice and WT were evaluated at 2, 4, 6 and 8 months of age. Tau-P301L male mice (*n* = 12) were not able to discriminate the novel from the familiar object at 6 months of age and Tau-P301L female mice (*n* = 7) at 4 months of age. Both WT male (*n* = 12) and female mice (*n* = 7) were able to discriminate the novel object. The one sample *t*-test against 0% was used to evaluate the missing discrimination from the novel object. *: *p* < 0.05, **: *p* < 0.01, ***: *p* < 0.001 and ****: *p* < 0.0001. Data are given as mean ±SEM.

**Figure 6:**
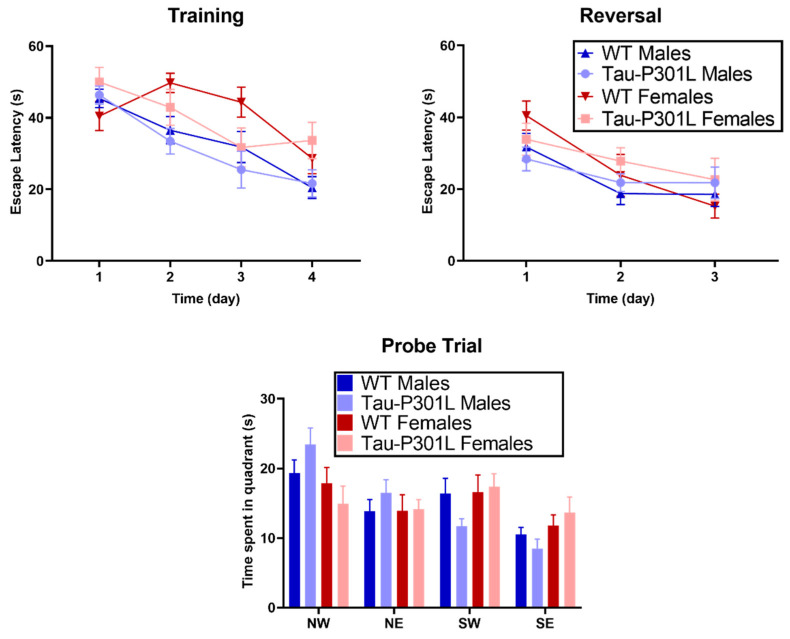
Tau-P301L mice did not have any deficits in the Morris Water Maze (MWM). Tau-P301L and WT mice were evaluated at 8 months of age. In the training and reversal test, both Tau-P301L mice (males: *n* = 12; females: *n* = 7) and WT mice (males: *n* = 12; females: *n* = 7) spent a similar amount of time to find the platform throughout the days. In the probe trial, both Tau-P301L mice and WT male mice explored the target quadrant similarly (NW). Mixed effect and two-way ANOVA were used for analysis, respectively. Data are given as mean ±SEM.

In order to analyze the development of possible cognitive deficits, several behavioral tests were performed. In the NOR, Tau-P301L mice were not able to discriminate between the novel and familiar object beginning at 6 months of age (Figure 5) (one sample *t*-test against 0%, 2 months: *p* = 0.0001; 4 months: *p* = 0.0045; 6 months: *p* = 0.2078 and 8 months: *p* = 0.1365). WT male mice were able to discriminate the novel from the familiar object at all analyzed ages significantly (one sample *t*-test against 0%, 2 months: *p* = 0.0001; 4 months: *p* < 0.0001; 6 months: *p* = 0.0157 and 8 months: *p* = 0.0153). Tau-P301L female mice did not discriminate the novel from the familiar object at 4 months of age (Figure 6) (one sample *t*-test against, 2 months: *p* = 0.0266; 4 months: *p* = 0.1221; 6 months: *p* = 0.1945 and 8 months: *p* = 0.1293) unlike the WT female mice (one sample *t*-test against, 2 months: *p* = 0.0037; 4 months: *p* = 0.0009; 6 months: *p* = 0.0562 and 8 months: *p* = 0.0187). In summary, since Tau-P301L mice did not significantly explore the novel object more, they had deficits in the recognition memory beginning at 6 (males) and 4 (females) months of age. 

No cognitive deficits were detectable, neither in the T-maze spontaneous alternation (Figure S5), nor in the contextual and cued fear conditioning within the here-analyzed ages (Figure S6). Furthermore, no differences could be detected between Tau-P301L and WT mice in the MWM. During the four days of training, all tested mice showed similar escape latencies (Figure 6). In the probe trial, all genotypes spent a similar amount of time in the target quadrant (NW). Moreover, no difference was detectable between Tau-P301L mice, neither between males nor between females. During the reversal trial, Tau-P301L mice and non-transgenic mice spent a similar amount of time to find the platform. Overall, Tau-P301L mice did not have any cognitive deficits in the MWM at the age of 8 months. 

### 3.4. Tau-P301L Showed Distinct Tau Pathology in the Brain at 8 Months of Age

**Figure 7:**
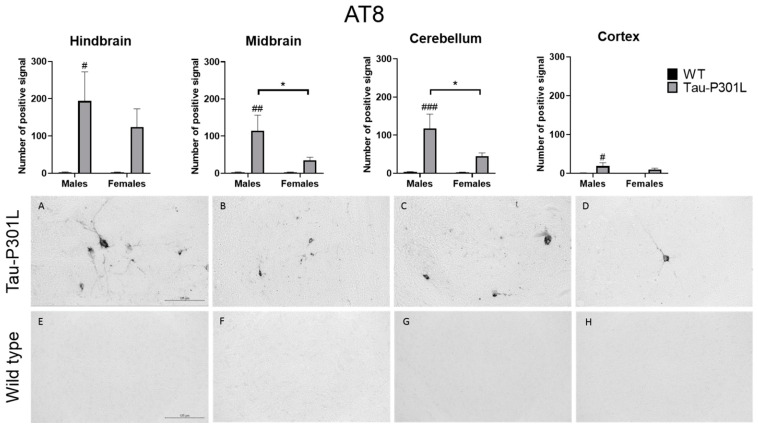
Tau-P301L mice show pathological Tau in different areas of the brain at 8 months of age. The phosphorylated Tau was detected by AT8 antibody. The hindbrain (**A**,**E**), cortex (**D**,**H**), midbrain (**B**,**F**) and cerebellum (**C**,**G**) from Tau-P301L (**A**–**D**) and wild type (WT) male mice (**E**–**H**) were analyzed. The positive signal was counted at different regions of the brain using ImageJ software. A two-way ANOVA was used for analysis. *: *p* < 0.05; #: *p* < 0.05, ##: *p* < 0.01 and ###: *p* < 0.001 compared to sex-matched WT. Scale bar is 125 µm.

After performance of the MWM, the brains from all mice were collected. Regarding the histopathology, an AT8-positive signal was found at a significantly higher number in the brains of Tau-P301L male mice compared to WT male mice. AT8 antibody binds to pSer202 and pThr204 and the phosphorylation of this site increases with age [19]. Therefore, those phosphorylated sites occur mainly in PHF [31,32]. In Tau-P301L male mice, more pathological Tau is found compared to WT male mice in the hindbrain (two-way ANOVA; males: *p* = 0.0131), the midbrain (two-way ANOVA; males: *p* = 0.0032), the cortex (two-way ANOVA; males: *p* = 0.0318) and the cerebellum (two-way ANOVA; males: *p* = 0.0009) (Figure 7). Moreover, Tau-P301L male mice (*n* = 8) had more AT-8 positive signals than Tau-P30L female mice (*n* = 7) in the midbrain (two-way ANOVA; *p* = 0.0452) and the cerebellum (two-way ANOVA; *p* = 0.0412). Finally, Tau-P301L female mice did not have more pathological Tau compared to WT male mice in any analyzed brain region. 

**Figure 8:**
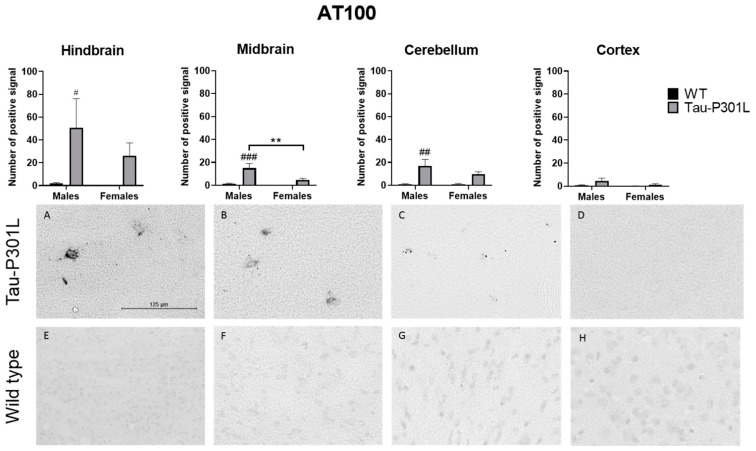
Tau-P301L mice had phosphorylated Tau in different areas of the brain at 8 months. The phosphorylated Tau was detected by AT100 antibody. The hindbrain (**A**,**E**), cortex (**D**,**H**), midbrain (**B**,**F**) and cerebellum (**C**,**G**) from Tau-P301L male (**A**–**D**) and wild type (WT) male mice (**E**–**H**) were analyzed. The positive signal was counted in different regions of the brain using ImageJ software. Two-way ANOVA and Multiple *t*-test were used for analysis. **: *p* < 0.01; #: *p* < 0.05, ##: *p* < 0.01 and ###: *p* < 0.001 compared to sex-matched WT. Scale bar is 125 µm.

Using the AT100 antibody that recognizes pSer214 and pThr212, which are only present in PHF [33] (Figure 8), it was found that Tau-P301L male mice had increased AT100 positive signal in the midbrain (two-way ANOVA; males: *p* = 0.0004), the hindbrain (two-way ANOVA; males: *p* = 0.0449) and the cerebellum (two-way ANOVA; males: *p* = 0.0024) compared to WT male mice. However, in the cortex, the number of positive signals was not significantly different from WT male mice. Regarding the sex, Tau-P301L male mice had an increased amount of AT100 positive signal only in the midbrain compared to Tau-P301L female mice (two-way ANOVA; *p* = 0.0083). Taken together, those regions with both AT100 and AT8 positive signal are mainly responsible for the motor coordination response and this could be an explanation for the motor deficits observed in the Tau-P301L male mice and not in the female mice. 

More specifically, pathological Tau is present throughout different nuclei in the hindbrain, especially in the locus coeruleus (LC), pontine reticular nuclei, vestibular nucleus (medial and spinal) and reticular nuclei (parvicellular and intermediate). In the midbrain, the nuclei with Tau pathology were found in the vestibular tegmental area, substantia nigra reticular, periaqueductal gray (PAG), midbrain reticular nuclei and superior colliculus. In the cerebellum, the main region where pathological Tau is present is the interposed nucleus. The pathological Tau observed in those regions were AT8- and AT100-positive, but as expected, more AT8 signal was observed compared to AT100. In the striatum, olfactory bulb and hippocampus, neither AT8 nor AT100 signal was detected; therefore, there is no pathological Tau in those regions.

Regarding the correlation between individuals, there is a clear relationship between the presence of Tau phosphorylation and the outcome of the behavioral test. Unfortunately, it was not possible to observe a statistically significant correlation between the results of the behavioral tests and the AT8/100 staining (Table S2).

Regarding neuronal loss, Tau-P301L male mice had fewer neurons in the hindbrain compared to WT (Table 3). The neurodegeneration was detected in the same region where the presence of AT8-positive signals but not AT100-positive signals was abundant. One could speculate that, since NFTs are mainly present intracellularly, the neuronal death in the hindbrain is inversely correlated with AT100 positive signal. Therefore, the increase of neuronal death would explain the low amount of AT100 positive signal in the hindbrain. No decrease of neurons was observed in Tau-P301L female mice in any region. In contrast, the reactive astrocytes and microglia were not increased in any brain region of Tau-P301L mice. 

## 4. Discussion

Translational research is essential to understand the mechanisms of diseases and mouse models play an important role in this context. Even though mouse models have several limitations, they are still the most complete option to be used in basic and preclinical research of neurodegenerative diseases [34]. For this reason, it is essential to characterize different mouse models down to the smallest details in order to obtain the most accurate translation and correlation to the corresponding human disease. In most characterization studies, only a few aspects of the phenotype are investigated and often only single ages are analyzed, e.g., when the first phenotypic differences are detectable, which might give limited information regarding the model. In this study, we focused on a longitudinal characterization study of the Tau-P301L mouse model, which was first described by Terwel and colleagues [19].

In summary, we showed that Tau-P301L mice had behavioral alterations in different behavioral tests probably due to the presence of pathological Tau in different brain regions. In the habituation/dishabituation olfactory test, Tau-P301L male mice spent less time smelling the newly presented aromas compared to WT male mice at 6 months of age. In the SHIRPA test, Tau-P301L mice had phenotypic alterations starting at 4 months of age. Moreover, the males had more prominent deficits compared to the females, especially regarding the motor alterations. In the modified pole test, Tau-P301L male mice had motor deficits demonstrated by a higher score compared to WT mice starting at 6 months of age. In the open field test, Tau-P301L male mice also had motor deficits, since they were slower, travelled less distance and explored less in an age-dependent manner compared to WT mice starting at 2 months of age. The Tau-P301L female mice did not show any of those alterations; therefore, one can assume they did not develop any motor deficits. Regarding the cognitive deficits, Tau-P301L male mice were not able to discriminate the novel from the familiar object in the NOR from 6 months of age. Moreover, Tau-P301L female mice did not discriminate the novel object from the familiar object at 4 months of age. Therefore, the Tau-P301L mouse model also displayed cognitive deficits. Those alterations can be explained by the presence of pathological Tau (AT8 and AT100 positive signal) in the hindbrain, cerebellum and midbrain, in which the latter ones are more pronounced in the Tau-P301L male mice than in female mice. The presence of pathological Tau induced neurodegeneration in the hindbrain in Tau-P301L male mice. Interestingly, the decrease of neurons seems to occur after the increase of AT8 positive signaling the hindbrain, but an increase of AT100 positive signal was not observed. Since AT100 antibody detects later stages of pathological Tau, one might speculate that the lack of increase of AT100 positive signal in the hindbrain might be due to the neuronal death of those neurons, which had pathological Tau. Finally, no increased activated astrocytes and microglia were observed in this study (Table 3); therefore, pathological Tau does not seem to induce the activation of astrocytes and microglia in Tau-P301L mice brain. 

In the present study, Tau-P301L mice did not show alterations on the rotarod at any analyzed ages, similar to the results published in previous studies [19,35]. In one study [35], it was described that Tau-P301L male mice on a C57BL/6J background showed phenotypic alterations at early ages (2 to 5 months of age) in some behavioral tests. Corroborating to the present study, Tau-P301L male mice did not develop any deficits at 4 months of age in the nesting and marble burying test. Moreover, we were able to demonstrate that Tau-P301L mice do not develop any deficits as late as 8 months of age in those tests. In the open field test, Tau-P301L mice travelled less as early as 2 months of age. This effect can be observed up to the age of 8 months. Again, these results agree with the one published by Samaey et al. [35]. In contrast to the study published by Samaey et al. [35], we were not able to observe any difference between Tau-P301L and WT mice in the amount of time they explored the different zones (border, center and corner). 

Described for the first time, Tau-P301L male mice had phenotypic alterations in the SHIRPA test battery. Starting at 4 months of age, Tau-P301L mice developed postural changes described by a hunched back, mild deficits in the hanging behavior and some mice developed a loss of the postural reflex. Then, beginning at 6 months of age, Tau-P301L mice started to display clasping of the limbs, which can be considered a paralysis, as well as an abnormal gait described as a waddling walk. Those alterations progressed with age. This result contrasts with those shown by other groups, analyzing other mouse models of tauopathy, since they did not describe any differences in the SHIRPA compared to the WT [36,37]. Only one study described a similar result regarding the hanging behavior. In this study, it was shown that the motor skills of Tau58-2/B mice (Tau-P301L mutation) were so limited regarding this specific subtest that the mice could not perform the test adequately [38]. In the present study, we also conducted a modified pole test. We were able to show that the mice exhibit deficits in this test that become more pronounced with increasing age. The motor deficits in the modified pole test were also described for another mouse model of tauopathy, called SJLB mouse model [39]. Moreover, Tau-P301L mice had olfactory deficits in the habituation/dishabituation olfactory test at 6 months of age. This deficit was also observed in another Tau-P301L mouse model [40]. Regarding the histopathology, neither AT8- nor AT100-positive signals were detected in the olfactory bulb/cortex. Therefore, another pathophysiological mechanism might play a role in the olfactory deficits observed in this study. Another explanation for this alteration is that the olfactory deficits bear on cognitive deficits, so one might speculate that Tau-P301L male mice were not able to recognize the new aroma.

The pathology described for this Tau-P301L mouse model is similar to the same and other mouse models with this specific mutation [19,20,21,41,42]. Therefore, the strain background and the used promoter do not seem to have any influence on the appearance of NFTs in the brains. However, the presence of NFTs can occur in different brain regions since the pR5 mouse model also shows tauopathy in the hippocampus [41], which is not observed in the mouse model from this study. The Tau pathology in Tau-P301L mice mainly occurs in the brainstem and might be an explanation for the behavioral deficits. The presence of pathological Tau in the brainstem, especially in SNr [43,44,45,46,47] and superior colliculus [48,49], can be related to motor deficits in Tau-P301L male mice. Moreover, the lack of NFTs in Tau-P301L female mice in those regions can be also related to the lack of motor deficits. Sex dimorphism is observed in other transgenic mouse models, but the results are contradictory [50]. Therefore, more experiments are needed in order to understand these sex-related differences more precisely.

Regarding the cognitive deficits, both Tau-P301L female and male mice developed deficits in the NOR that are in line with the Tau aggregation in the LC, since it also plays a role in cognition, and NFTs in this region induce cognitive deficits [51,52]. Tau-P301L mice did not have any deficits in the MWM, T-Maze and contextual fear conditioning, probably due to the lack of NFTs in the hippocampus, which play a main role in processing of the spatial memory [53]. Tau-P301L mice also did not show cognitive deficits in the cued fear conditioning, probably also due to the lack of NFTs in the amygdala, which is the region that processes fear memory [54].

Sex differences in neurodegenerative diseases are observed in both animals and humans. In humans, women have a higher probability to develop AD than men as well as developing a more severe pathology [55,56]. In AD mice, Aβ levels are also higher and cognitive deficits are more prominent in females. Regarding Tau pathology, not much information is available about transgenic models. In the present study, Tau-P301L male mice had motor deficits compared to females, even though both sexes had cognitive deficits. Another study also demonstrated motor deficits in the Tau-P301S males and later cognitive deficits compared to females, but a similar tau pathology in the brain [57]. In a triple transgenic mouse model, which develops both Aβ plaques and NFTs, 3xTg-AD females had a higher amount of Tau pathology and cognitive deficits compared to males. This discrepancy might be due to the age of the tested mice, since AD mice develop the alterations later than Tau mice [58]. Additionally, female reproductive senescence is reached at 12 months of age, when estrogen levels are decreased [59]. Estrogen is known to have neuroprotective effects and its decrease might explain the severity of tau pathology in the 3xTg-AD females [60,61,62,63]. In this study, reproductive active females were evaluated and the estrogen levels might explain the milder Tau pathology in females. Still, it is important to highlight that the comparison of different models must be done with caution, since each model has different behavioral and physiopathological outcomes. Moreover, more studies are needed to further explain the remarkable sex differences in the Tau-P301L mouse model, as observed in this study. 

In conclusion, this longitudinal study demonstrates that Tau-P301L mice have alterations due to the presence of pathological Tau in the brain that agree with age and are sex-dependent. Tau-P301L male mice had olfactory deficits, motor deficits and increased Tau pathology in the brain. None of those alterations were observed in Tau-P310L female mice. Both sexes, however, had phenotypic alterations in the SHIRPA test battery and cognitive deficits in the NOR. It is possible to determine that the disease onset in the males occurs as early as 2 months of age regarding the motor deficits and 6 months of age regarding the cognitive deficits. 

## Figures and Tables

**Table 1:** Evaluated Parameter on the SHIRPA test.

Parameters	Description
Restlessness	Difficulty staying in one body position for an extended period of time
Apathy	Motionless and lowered head
Stereotyped behaviour	
Convulsion	
Abnormal body carriage	Body posture
Alertness	Response to object proximity
Abnormal gait	Uncommon walk, e.g., paddling, waddling, running
Startle response	Response to an acoustic signal
Loss of righting reflex	Time when the mouse return to standing position when turned on its back
Touch response	
Pinna reflex	
Cornea reflex	
Forelimb placing reflex	Response to stretch their front paws when hanged in proximity to the surface
Hanging behaviour	Mouse stays on the rod or falls
Pain response	Response to tail pinch
Grooming	Overall fur condition

**Table 2:** Tau-P301L mice showed phenotypic alterations in different evaluated parameters in the SHIRPA test starting at 4 months of age.

Parameters	Phenotypic Alterations
Restlessness	No alterations
Apathy	No alterations
Stereotyped behavior	No alterations
Convulsion	No alterations
Abnormal body carriage	Hunchback
Alertness	No alterations
Abnormal gait	Waddling walk and slower compared to WT
Startle response	No alterations
Loss of righting reflex	Some Tau-P301L mice have light loss of righting reflex
Touch response	Less responsive to touch than WT
Pinna reflex	No alterations
Cornea reflex	No alterations
Forelimb placing reflex	Paralysis (“Clasping”) of the limbs
Hanging behavior	Tau-P301L male mice fall faster from the rod than WT male mice
Pain response	No alterations
Grooming	The Tau-P301L male mice have very good fur condition compared to WT

**Table 3:** Neuronal loss and gliosis in Tau-P301L mice in different brain regions.

Staining	Brain Region	WT		Tau-P301L		Significance
		Males	Females	Males	Females	
NeuN (Spot Count)	Hindbrain	1434.7 ± 111.3	1307.6 ± 208.9	817.1 ± 181.7	1286.0 ± 246.4	WT males vs. Tau-P301L males (*p* = 0.012)
	Midbrain	1376.8 ± 184.5	1487.8 ± 108.9	1191.9± 139.9	1474.3 ± 237.6	n.s
	Cortex	3545.1 ± 197.0	2879.1 ± 262.8	4141.1 ± 210.7	3651.3 ± 285.7	n.s
	Cerebellum	1834.0 ± 83.6	1259.0 ± 123.5	2060.1 ± 158.2	1461.1 ± 187.0	n.s
GFAP (Stained Area)	Hindbrain	27.0 ± 1.0	29.3 ± 1.5	31.5 ± 3.6	33.4 ± 1.1	n.s
	Midbrain	18.6 ± 2.8	21.0 ± 3.3	19.9 ± 2.8	31.2 ± 2.1	n.s
	Cortex	15.7 ± 2.7	23.4 ± 3.1	21.7 ± 4.4	31.8 ± 1.7	n.s
	Cerebellum	8.5 ± 1.3	11.0 ± 1.8	11.8 ± 1.6	15.4 ± 1.0	n.s
CD11b (Stained Area)	Hindbrain	6.7 ± 0.9	5.6 ± 0.4	6.5 ± 0.6	5.7 ± 0.8	n.s
	Midbrain	6.0 ± 0.4	5.2 ± 0.6	5.5 ± 0.7	5.1 ± 0.5	n.s
	Cortex	6.0 ± 0.5	5.9 ± 0.6	6.9 ± 0.9	6.0 ± 0.7	n.s
	Cerebellum	6.5 ± 0.4	4.9 ± 0.7	6.6 ± 0.3	5.8 ± 0.3	n.s

Quantification of activated astrocytes (GFAP), reactive microglia (CD11b) and neuronal nuclei (NeuN) of 8-month-old Tau-P301L (Tau-P301L) and wild type (WT) mice. The spot count analysis per selected area analysis was done in different brain regions (cortex, cerebellum, midbrain and hindbrain), resulting in a significant decrease of neurons in the Tau-P301L males’ hindbrain. Analysis of gliosis, evaluated as stained area, revealed no differences between groups in different regions. Not statistically significant is represented by n.s.

## Data Availability

All data from the study are available in this manuscript.

## Supplementary Materials

The following are available online at https://www.mdpi.com/article/10.3390/biomedicines9091160/s1, Figure S1: Tau-P301L mice had similar weight compared to WT mice; Figure S2: Tau-P301L mice had similar performance in the nesting and marble burying test compared to WT mice; Table S1: Tau-P301L mice (Tau) had increased scores in different parameters compared to WT starting with 4 months of age in the SHIRPA test battery; Figure S3: Tau-P301L mice had similar performance in the Rotarod test compared to WT mice; Figure S4: Tau-P301L mice spent similar amount of time in the border and center of the open field compared to WT mice; Figure S5: Tau-P301L mice had similar performance in the T-maze spontaneous alternation compared to WT mice; Figure S6: Tau-P301L mice froze similarly compared to WT mice in the cued and contextual fear conditioning; Table S2: Correlation between behavioral tests and AT8 as well as AT100 staining.
