# Supplementary material for: Sex-Related Motor Deficits in the Tau-P301L Mouse Model
Source: Biomedicines. 2021 Sep 4;9(9):1160. doi: 10.3390/biomedicines9091160 (PMC8471835; doi:10.3390/biomedicines9091160)
Supplement: Supplementary file 1 [file biomedicines-09-01160-s001.zip › biomedicines-1340021-supplementary.pdf]

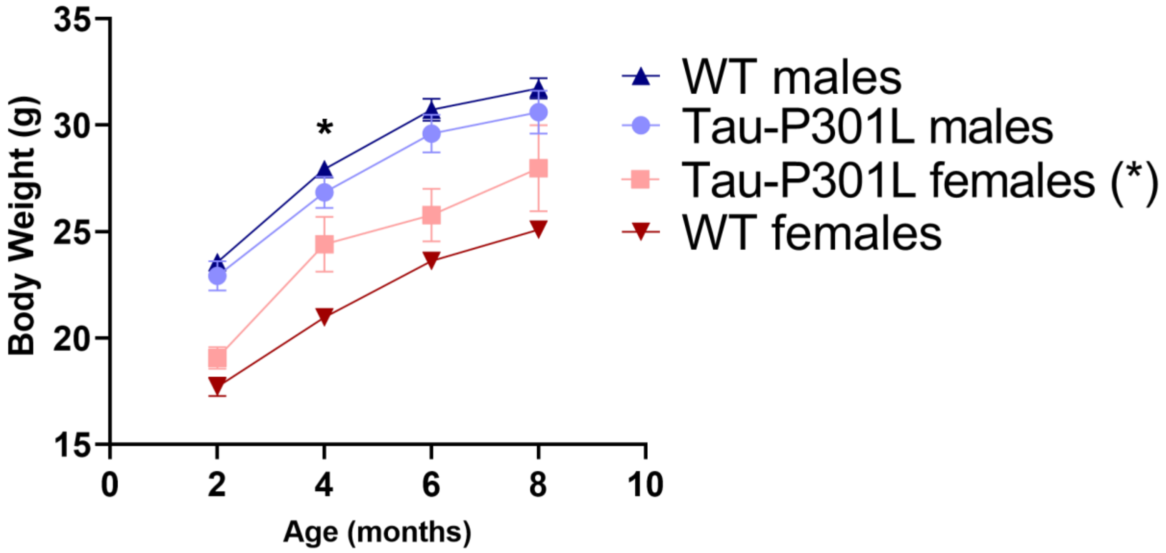

Figure S1*:* Tau-P301L mice had similar body weight compared to WT mice. Tau-P301L male mice (n = 12) did not differ from age-matched WT male mice (n = 12) in the body weight. Tau-P301L female mice (n = 7) had higher body weight only at 4 months of age compared to age-matched WT female mice (n = 7; *: p = 0.045). Two- way ANOVA and post hoc Tukey's was used as statistical analysis. Data given as mean ± SEM.

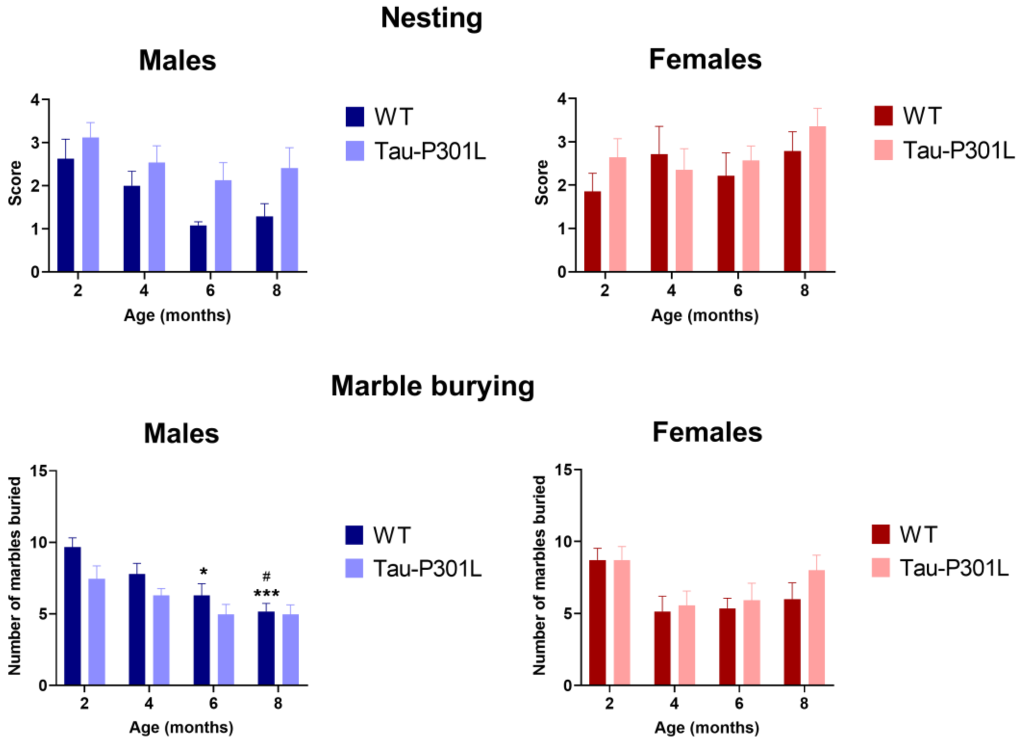

Figure S2*:* Tau-P301L mice had similar performance in the nesting behavior and marble burying test compared to WT mice. No difference was observed between Tau-P301L male mice (n = 12) and age-matched WT male mice (n = 12) regarding the nesting behavior and numbers of marbles buried. Performance of Tau-P301L female mice (n = 7) and age-matched WT female mice (n = 7) was also similar in both tests. Two- way ANOVA was used as statistical

analysis. Data given as mean± SEM. *: p = 0.0127, ***: p = 0.0002 compared to 2 months genotype-matched. #: p = 0.0236 compared to 4 months genotype-matched.

Table S1: Tau-P301L mice (Tau) had increased scores in different parameters compared to WT starting with 4 months of age in the SHIRPA test battery. Data given as mean± SEM.

**Parameters**	**Score**															
	2 Months				4 Months				6 Months				8 Months			
	Male		Female		Male		Female		Male		Female		Male		Female	
	WT	Tau	WT	Tau	WT	Tau	WT	Tau	WT	Tau	WT	Tau	WT	Tau	WT	Tau
**Restlessness**	0.0 ± 0.0	0.0 ± 0.0	0.0 ± 0.0	0.0 ± 0.0	0.0 ± 0.0	0.0 ± 0.0	0.0 ± 0.0	0.0 ± 0.0	0.1 ± 0.1	0.1 ± 0.1	0.0 ± 0.0	0.0 ± 0.0	0.0 ± 0.0	0.0 ± 0.0	0.0 ± 0.0	0.0 ± 0.0
**Apathy**	0.0 ± 0.0	0.0 ± 0.0	0.0 ± 0.0	0.0 ± 0.0	0.0 ± 0.0	0.0 ± 0.0	0.0 ± 0.0	0.0 ± 0.0	0.0 ± 0.0	0.0 ± 0.0	0.0 ± 0.0	0.0 ± 0.0	0.0 ± 0.0	0.0 ± 0.0	0.0 ± 0.0	0.0 ± 0.0
**Stereotyped behavior**	0.0 ± 0.0	0.0 ± 0.0	0.0 ± 0.0	0.0 ± 0.0	0.0 ± 0.0	0.0 ± 0.0	0.0 ± 0.0	0.0 ± 0.0	0.0 ± 0.0	0.0 ± 0.0	0.0 ± 0.0	0.0 ± 0.0	0.0 ± 0.0	0.0 ± 0.0	0.0 ± 0.0	0.0 ± 0.0
**Convulsion**	0.0 ± 0.0	0.0 ± 0.0	0.0 ± 0.0	0.0 ± 0.0	0.0 ± 0.0	0.0 ± 0.0	0.0 ± 0.0	0.0 ± 0.0	0.0 ± 0.0	0.0 ± 0.0	0.0 ± 0.0	0.0 ± 0.0	0.0 ± 0.0	0.0 ± 0.0	0.0 ± 0.0	0.0 ± 0.0
**Abnormal body carriage**	0.0 ± 0.0	0.0 ± 0.0	0.0 ± 0.0	0.0 ± 0.0	0.3 ± 0.1	0.8 ± 0.1	0.0 ± 0.0	0.7 ± 0.2	0.3 ± 0.1	0.9 ± 0.1	0.1 ± 0.1	0.9 ± 0.1	0.1 ± 0.1	0.5 ± 0.2	0.1 ± 0.0	0.4 ± 0.2
**Alertness**	0.0 ± 0.0	0.0 ± 0.0	0.0 ± 0.0	0.0 ± 0.0	0.0 ± 0.0	0.0 ± 0.0	0.1 ± 0.1	0.1 ± 0.1	0.0 ± 0.0	0.2 ± 0.1	0.1 ± 0.1	0.0 ± 0.0	0.1 ± 0.1	0.0 ± 0.0	0.1 ± 0.1	0.0 ± 0.0
**Abnormal gait**	0.0 ± 0.0	0.0 ± 0.0	0.0 ± 0.0	0.0 ± 0.0	0.1 ± 0.0	0.7 ± 0.2	0.0 ± 0.0	0.4 ± 0.2	0.2 ± 0.1	1.3 ± 0.2	0.0 ± 0.0	0.3 ± 0.2	0.1 ± 0.1	0.7 ± 0.1	0.0 ± 0.0	0.4 ± 0.2
**Startle response**	0.2 ± 0.1	0.3 ± 0.1	0.1 ± 0.1	0.1 ± 0.1	0.4 ± 0.1	0.3 ± 0.1	0.0 ± 0.0	0.1 ± 0.1	0.3 ± 0.1	0.3 ± 0.1	0.3 ± 0.3	0.1 ± 0.1	0.0 ± 0.0	0.0 ± 0.0	0.0 ± 0.0	0.0 ± 0.0
**Loss of righting reflex**	0.0 ± 0.0	0.0 ± 0.0	0.1 ± 0.1	0.9 ± 0.1	0.3 ± 0.1	0.7 ± 0.2	0.1 ± 0.1	0.7 ± 0.4	0.3 ± 0.1	0.5 ± 0.2	0.0 ± 0.0	0.9 ± 0.4	0.0 ± 0.0	0.2 ± 0.1	0.0 ± 0.0	0.4 ± 0.2
**Touch response**	0.0 ± 0.0	0.2 ± 0.1	0.0 ± 0.0	0.0 ± 0.0	0.1 ± 0.1	0.7 ± 0.2	0.1 ± 0.1	0.3 ± 0.2	0.1 ± 0.1	0.2 ± 0.1	0.1 ± 0.1	0.1 ± 0.1	0.0 ± 0.0	0.1 ± 0.1	0.0 ± 0.0	0.1 ± 0.1
**Pinna reflex**	0.0 ± 0.0	0.0 ± 0.0	0.0 ± 0.0	0.0 ± 0.0	0.1 ± 0.1	0.3 ± 0.1	0.0 ± 0.0	0.1 ± 0.1	0.1 ± 0.1	0.0 ± 0.0	0.0 ± 0.0	0.1 ± 0.1	0.0 ± 0.0	0.0 ± 0.0	0.0 ± 0.0	0.0 ± 0.0
**Cornea reflex**	0.0 ± 0.0	0.0 ± 0.0	0.0 ± 0.0	0.0 ± 0.0	0.3 ± 0.1	0.3 ± 0.1	0.0 ± 0.0	0.0 ± 0.0	0.2 ± 0.1	0.2 ± 0.1	0.1 ± 0.1	0.3 ± 0.2	0.0 ± 0.0	0.1 ± 0.1	0.0 ± 0.0	0.0 ± 0.0
**Forelimb placing reflex**	0.1 ± 0.1	0.3 ± 0.1	0.0 ± 0.0	0.1 ± 0.1	0.1 ± 0.1	0.3 ± 0.1	0.0 ± 0.0	0.0 ± 0.0	0.1 ± 0.1	0.4 ± 0.1	0.1 ± 0.1	0.0 ± 0.0	0.2 ± 0.1	0.8 ± 0.1	0.1 ± 0.1	0.6 ± 0.3
**Hanging behavior**	0.2 ± 0.1	0.4 ± 0.1	0.0 ± 0.0	0.3 ± 0.1	0.1 ± 0.1	0.8 ± 0.3	0.0 ± 0.0	0.0 ± 0.0	0.3 ± 0.1	0.8 ± 0.3	0.9 ± 0.4	0.9 ± 0.3	0.3 ± 0.1	0.8 ± 0.3	0.7 ± 0.2	0.7 ± 0.3
**Pain response**	0.1 ± 0.1	0.1 ± 0.1	0.0 ± 0.0	0.0 ± 0.0	0.3 ± 0.1	0.3 ± 0.1	0.0 ± 0.0	0.3 ± 0.1	0.1 ± 0.1	0.4 ± 0.1	0.1 ± 0.1	0.4 ± 0.2	0.0 ± 0.0	0.1 ± 0.1	0.0 ± 0.0	0.0 ± 0.0
**Grooming**	0.0 ± 0.0	0.0 ± 0.0	0.0 ± 0.0	0.0 ± 0.0	0.2 ± 0.1	0.1 ± 0.1	0.0 ± 0.0	0.1 ± 0.1	0.3 ± 0.2	0.3 ± 0.1	0.0 ± 0.0	0.1 ± 0.1	0.0 ± 0.0	0.2 ± 0.1	0.0 ± 0.0	0.1 ± 0.1
**Score Sum**	**0.5 ± 0.2**	**1.3 ± 0.3**	**0.3 ± 0.3**	**1.4 ± 0.4**	**2.1 ± 0.2**	**5.2 ± 0.6**	**0.4 ± 0.3**	**3.0 ± 0.4**	**2.1 ± 0.3**	**5.4 ± 0.6**	**2.0 ± 0.4**	**4.1 ± 0.7**	**0.7 ± 0.2**	**3.3 ± 0.4**	**1.1 ± 0.3**	**2.7 ± 0.6**

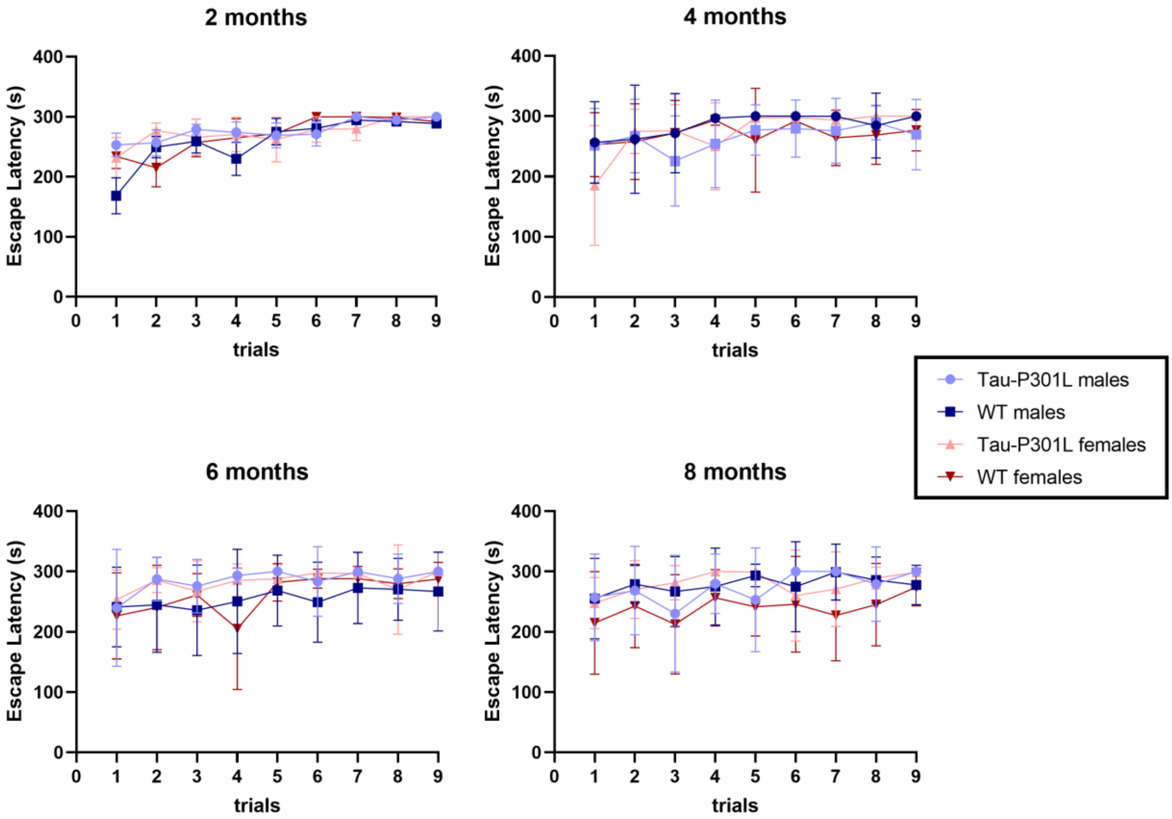

Figure S3*:* Tau-P301L mice had similar performance in the Rotarod test compared to WT mice. No motor deficits were observed in Tau-P301L male mice (n = 12) compared to age-matched WT male mice (n = 12), as well as in Tau-P301L female mice (n = 7) compared to age-matched WT female mice (n = 7). Two-way ANOVA was used as statistical analysis. Data given as mean± SEM.

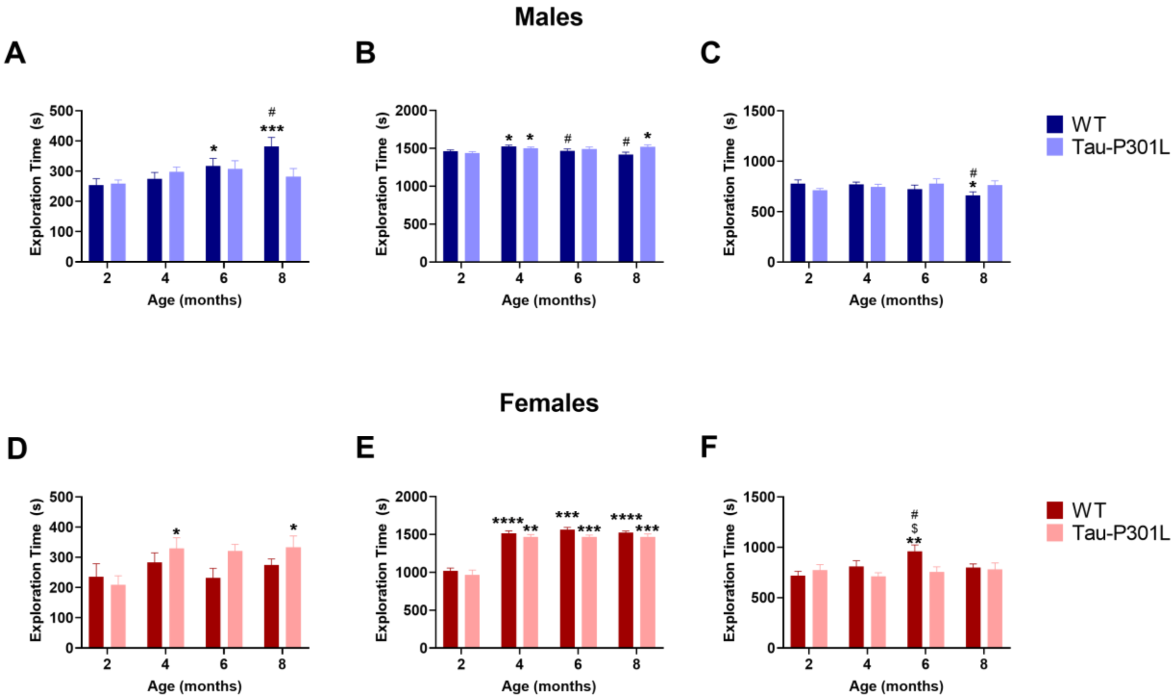

Figure S4: Tau-P301L mice spent similar amount of time in the border and center of the open field compared to WT mice. No difference was observed in Tau-P301L male mice (n = 12) compared to age-matched WT male mice (n = 12) as well as in Tau-P301L female mice (n = 7) compared to age-matched WT female mice (n = 7), regarding the time spent in the center (A and D), border (B and E) and corner (C and F) of the open field arena. The two- way ANOVA was used as statistical analysis. Data given as mean ± SEM.*: $p < 0.05$, **: $p < 0.01$, ***: $p = 0.001$ and ****: $p < 0.0001$ compared to 2 months genotype-matched. #: $p < 0.05$ compared to 4 months genotype-matched. $: $p < 0.05$ compared to 8 months genotype-matched.

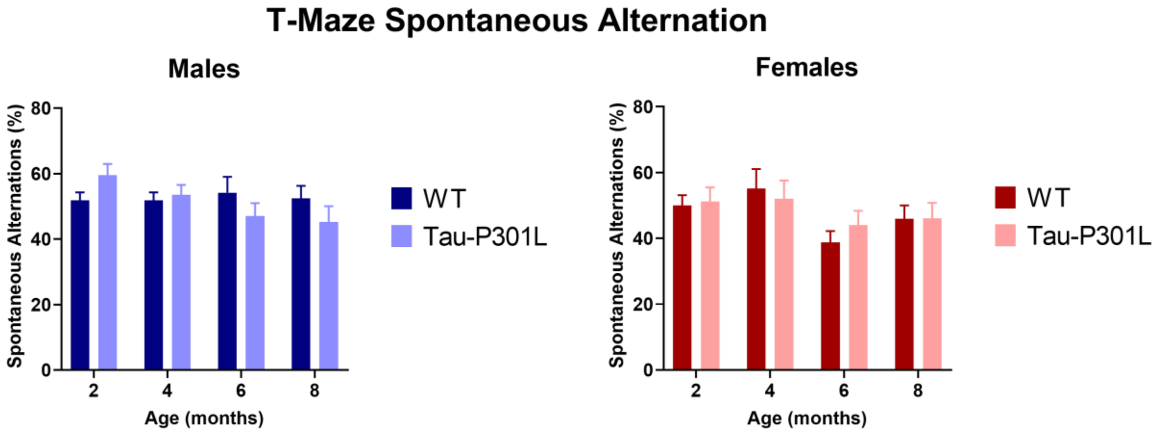

Figure S5: Tau-P301L mice had similar performance in the T-maze spontaneous alternation compared to WT mice. Tau-P301L male mice (n = 12) alternated similarly to age-matched WT male mice (n = 12), as well as Tau-P301L female mice (n = 7) compared to age-matched WT female mice (n = 7) in the T-maze spontaneous alternation. Two-way ANOVA was used as statistical analysis. Data given as mean± SEM.

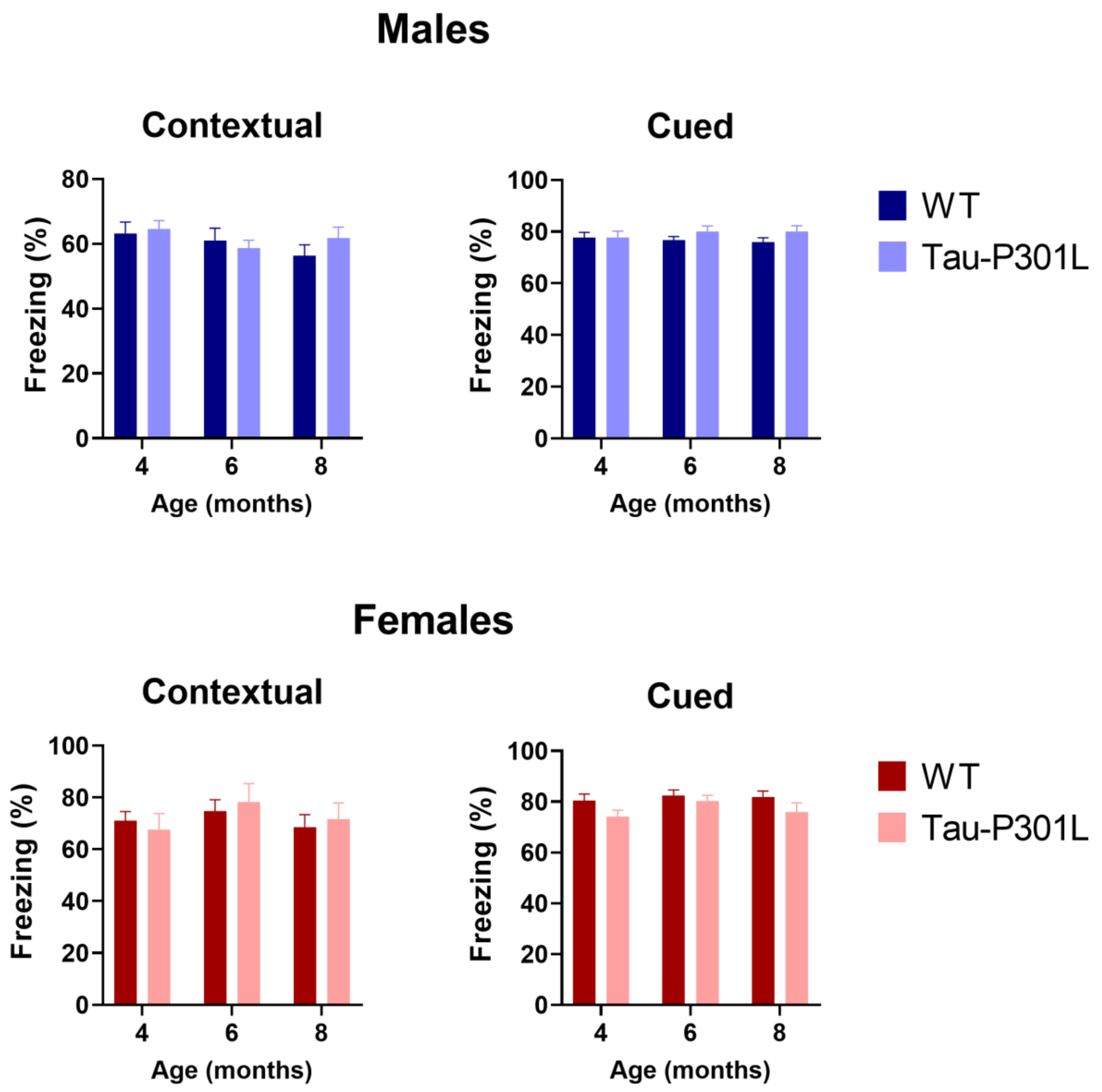

Figure S6: Tau-P301L mice froze similarly compared to WT mice in the cued and contextual fear conditioning. Tau-P301L male mice (n = 12) had similar percentage of freezing compared to age-matched WT male mice (n = 12), as well as in Tau-P301L female mice (n = 7) compared to age-matched WT female mice (n = 7) in the cued and contextual fear conditioning. Two- way ANOVA was used as statistical analysis. Data given as mean± SEM.

Table S2: Correlation between behavioral tests and AT8 as well as AT100 staining.

	AT8				AT100			
	Hindbrain	Midbrain	Cerebellum	Cortex	Hindbrain	Midbrain	Cerebellum	Cortex
**SHIRPA**	r = -0.42<br>p = 0.15	r = -0.013<br>p = 0.5	r = -0.21<br>p = 0.31	r = 0.7<br>p = 0.03	r = 0.33<br>p = 0.43	r = 0.32<br>p = 0.44	r = -0.25<br>p = 0.55	r = 0,1<br>p = 0.81
**Pole**	r = 0.39<br>p = 0.17	r = 0.54<br>p = 0.085	r = 0.54<br>p = 0.085	r = -0.15<br>p = 0.36	r = 0.12<br>p = 0.78	r = -0.41<br>p = 0.31	r = -0.13<br>p = 0.76	r = -0.47<br>p = 0.24
**OF**	r = -0.43<br>p = 0.29	r = -0.14<br>p = 0.73	r = -0.26<br>p = 0.54	r = -0.29<br>p = 0.48	r = -0.38<br>p = 0.35	r = -0.24<br>p = 0.56	r = -0.31<br>p = 0.45	r = -0.15<br>p = 0.72
**NOR**	r = -0.22<br>p = 0.6	r = -0.17<br>p = 0.68	r = -0.25<br>p = 0.56	r = 0.25<br>p = 0.56	r = -0.1<br>p = 0.81	r = -0.02<br>p = 0.96	r = -0.12<br>p = 0.77	r = -0.03<br>p = 0.94

r = Spearman correlation coefficient
OF = open field test (locomotion)
NOR = novel object recognition (discrimination index)
